# Influence of the Type of Binder Used in the Treatment of Cotton Fabric with Montmorillonite Particles on the Release of Negative Ions

**DOI:** 10.3390/polym14224945

**Published:** 2022-11-16

**Authors:** Margaux Carette, Jaime Gisbert-Payá, Lucía Capablanca, Eva Bou-Belda

**Affiliations:** 1 Faculty of Sciences, HO University College Ghent, Valentin Vaerwyckweg 1, 9000 Ghent, Belgium; 2 Department of Textile and Paper Engineering, Universitat Politècnica de València, Plaza Ferrándiz y Carbonell s/n, 03801 Alcoy, Spain

**Keywords:** functional textile, cotton fabric, NAI, healthy, smart textile

## Abstract

Throughout history, mineral clays have had a multitude of applications. With recent developments in the textile industry, they have been used for their antimicrobial properties. As a promising phyllosilicate with a negative layer charge, montmorillonite (MMT) was used in this work to treat cotton fabric to evaluate its ability to generate negative air ions (NAIs). The MMT was dispersed with varying binder concentrations. Resins of different composition (polyurethane or acrylic) was applied to cotton fabric by padding, and the negative ion count was measured. Two types of MMT with different characteristics were tested. Electronic microscopy (SEM) was used to study the presence of MMT particles on the cotton fabric surface, and the colors of the samples were tested. It was observed that the composition of the binder used had a significant influence on the number of negative ions released by the treated sample.

## 1. Introduction

Air ions are formed when gaseous molecules or atoms obtain a significantly high energy, that is, enough to eject an electron. Different sources, both natural and artificial, can be held responsible for the formation of electrically charged ions, including cosmic radiation, UV rays, corona discharge, shearing forces of water (Lenard effect), plant-based sources of energy, etc. [1].

Several experiments have indicated that exposure to negative ions is linked to a series of health benefits, such as a reduction in the severity of depression, a reduction in anxiety, and enhanced well-being (for instance, relief from hay fever, better sleep cycle, less aches and pains, and improved metabolism, among other benefits). It is said that NAIs affect the nervous and cardiovascular system as well as mental health, and although the effects on the nervous and cardiovascular system were not quantitatively evaluated, an improvement in symptoms in mood disorders has been clearly documented in several case studies. Though the measurement of mood in a study population is difficult and not completely standardized, a clear trend was noticed with NAI concentrations above 10^3^–10^5^ ions/cm^3^ [1,2].

Several literature sources mention fabric treatments to add different particles as an alternative source for NAI production [3]. In previous work, an increase in the amount of NAI released by fabric treated with montmorillonite particles was confirmed [4]. These types of alumina-silicate are phyllosilicates, a collection of silicates defined by their sheet-like structure. Because of their structure, they are capable of carrying a layer charge (positive or negative). In this study, the focus was placed on silicates that can carry a negative charge, as this study’s directive was the amelioration of overall human health using negative air ions.

Montmorillonite (MMT) is a type of inorganic natural clay mineral that typically consists of silicate (SiO_4_) tetrahedral and octahedral sheets arranged into a two-dimensional network structure with a layer charge of approximately 0.25–0.40. As isomorphous substitution in the sheets and the resulting cation addition to balance the excess charge make for varying chemical formulas, the exact formula for montmorillonite is never seen in nature. A general formula for montmorillonite can be found here, with ‘X’ indicating the amount of water in the mineral type [5]:

(Ca, Na, H) (Al, Mg, Fe, Zn)_2_ (Si, Al)_4_ O_10_(OH)_2_ · XH_2_O

The octahedral sheets that build up the crystal structure, together with tetrahedral sheets, are dioctahedral. Montmorillonite is known to have a layer charge of 0.25–0.40 and a high cation exchange capacity (CEC) of 70–110 meq/100 g [5].

Characterized as having a greater than 50% octahedral charge, its cation exchange capacity is due to isomorphous substitution of Mg^2+^ for Al^3+^ in the central alumina plane of the dioctahedral sheets. The substitution of lower valence cations in such instances leaves the nearby oxygen atoms with a net negative charge that can attract cations [6,7].

Textile substrates can be functionalized by providing new properties through various processes or treatments [8,9,10,11], and one of the most commonly used techniques is the incorporation of particles with certain properties that once present in the substrate, it acquires these same properties [12,13,14,15,16]. In this work, MMT particles, which have no affinity for the fiber [17], were added by a wet textile process, so it was necessary to use a resin that helped to adhere the particles to the surface of the fibers, and thus achieve greater durability and resistance to the daily wear and tear to which textile articles are subjected to throughout their life. The objective of this work was to study the influence of the nature of the resin, as well as the resin concentration used in the formulation, on the release of negative ions from the treated textile substrate. In the same way, a comparative study of the type of MMT used in the treatment was carried out, and all the results were compared with the untreated fabric. The treatments were carried out on a cotton fabric, and two resins, polyurethane and acrylic, were used as binding agents. In order to characterize the MMT particles and to determine the deposition of these particles on the fabric, scanning electron microscopy was used. The reflection spectrophotometer was used to measure the color of the fabrics and to evaluate the color change produced after the treatment. This parameter had to be taken into account to analyze the change in appearance caused by the compounds used in the treatment bath. In order to measure the number of ions released by the textile, an iometer was used.

## 2. Materials and Methods

A 100% twill cotton fabric with 210 g/m^2^ was used. The fabric had been desized, scoured and bleached.

Lurapre^®^ dispersion TX 6072 supplied by Archroma (Pratteln, Switzerland) was used as the polyurethane binder, and Resina center SFK/100 supplied by Color Center (Terrassa, Spain) was used as the acrylic binder.

Montmorillonite K10 (MMT K10) and Montmorillonite KSF (MMT KSF), both supplied by Sigma Andrich (St. Louis, MO, USA), were used as aluminasilicate particles. These particles show different characteristics that can influence the amount of NAI released from the treated fabric. In Table 1 the main characteristics are shown.

MMT K10 is an acid-activated and thermally treated montmorillonite; these modifications result in the partial destruction of the crystal structure, the leaching of the octahedral cations (Al or Mg) and a loss of swelling ability. Due to the leaching and gradual destruction of the octahedral sheet (sandwiched between tetrahedral sheets), K10 forms a highly porous substance with an increase in both surface area (220–270 m^2^/g) and pore diameter, and the development of a net negative surface charge [18]. Montmorillonite KSF (Sigma Aldrich, St. Louis, MO, USA) is also an acid-activated silicate, used without additional modification. This montmorillonite has a particle size range of 1–90 µm and a surface area of 20–40 m^2^/g, which is significantly lower than that of montmorillonite K10.

The textiles were treated using the padding method. A bath was prepared containing the silicate and binder diluted in water, in which the fabric was dipped. After passing through the pad bath, the fabric was guided through pad rolls to remove any excess solution. The pick-up obtained in all treated samples was 80–85%. After the padding process fabrics were dried for 24 h, they were cured in the oven at 120 °C for 10 min.

For every sample, a bath was prepared by adding 20 g/L of MMT into a solution of binding agent with three different concentrations, i.e., 1 g/L, 2.5 g/L and 5 g/L, respectively. For the sake of comparison, baths with only silicate and only binder were also prepared. In Table 2 formulations used in each treatment are shown.

The ZEISS AURIGA Compact model FESEM was used in order to characterize the particles and the treated fabrics. The fabric samples (6 mm × 6 mm) were placed on a disk using carbon tape and coated at a thickness of 1nm using Platinum Black. The images were procured by a secondary electron in-lens detector (SE2) located inside the electron column. This provides topography images of the sample surfaces with high resolution, with the best performance at low acceleration potentials (<5 kV).

The whiteness index was determined in the CIELAB color space from diffuse reflectance measurements according to ISO 105-J01:1997 and ISO 105-J02:1997, using the following CIE Formula (1):

(1) $$W_{10}=Y_{10}+800\left(0.3138-x_{10}\right)+1700\left(0.3310-y_{10}\right)$$

with:

$W_{10}$ = whiteness index value
$Y_{10}$ = tristimulus value for the sample
$x_{10},\;y_{10}$ = the colour coordinates

CIELAB color coordinates (L*, a*, b*) were calculated from the reflectance data for 10° observer and illuminant D65. The shift of the coordinates of the color in the color spaces L*, a*, and b*, based on the theory that color is perceived in terms of black–white (L), red–green (a), and yellow–blue (b), was summarized by the ΔE* value. The value of ΔE* represents the overall color difference between each treated sample and the standard (untreated sample).

Following the Gordian tube method described by the authors Roubal et al., the negative ion count was measured. The ION COUNTER COM-3200cd was used.

## 3. Results

### 3.1. MMT Characterization

**Figure 1:**
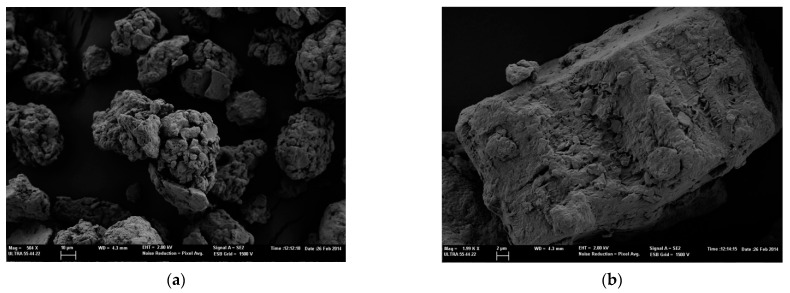
FESEM images of MMT KSF microparticles. (**a**) At 500× magnification and (**b**) at 2000× magnification.

Figure 1 shows two microphotographs at different magnifications in order to visualize the appearance, shape, and size of the micro-particles referenced as MMT KSF.

**Figure 2:**
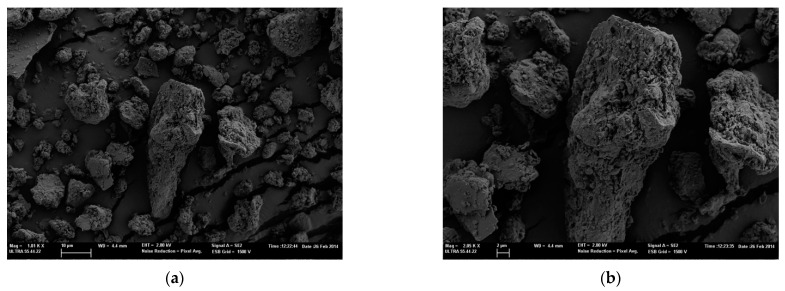
FESEM images of MMT K10 microparticles. (**a**) At 500× magnification and (**b**) at 2000× magnification.

Similarly, in Figure 2, images of silica particles MMT K10 are shown. The images of the two types of characterized nanoparticles show a wide range of size distribution, coinciding with the size distribution described by the supplier of the two silica particles, 1–40 µm and 1–90 µm of MMT K10 and MMT KSF.

### 3.2. Whiteness Index

**Figure 3:**
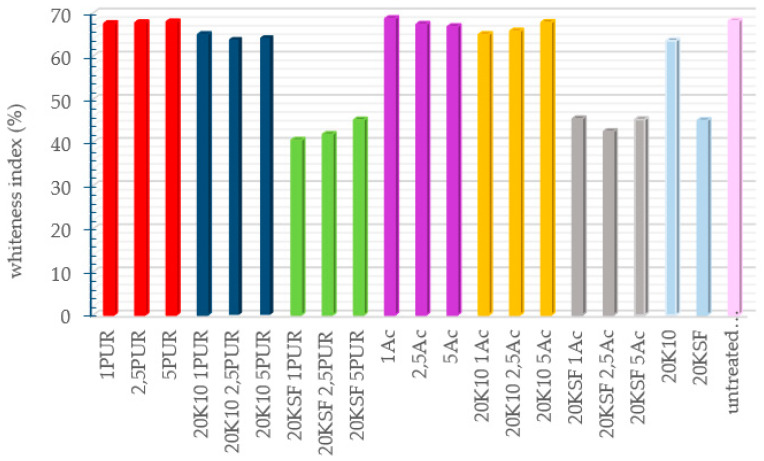
WI CIE results of the treated samples using different formulation.

Color measurements of the treated samples were performed in order to know the change of the cotton fabric’s appearance in terms of whiteness. In Figure 3, the results of the whiteness measurements of each of the untreated and treated fabrics are shown. Overall, it can be observed that the fabric samples treated with KSF show the most color change from an untreated sample. A significant difference is noticeable in all the graphs obtained by the spectrophotometer between the samples treated with MMT KSF (both with and without binder) and the other samples (almost 30%), while the samples treated with MMT K10 differed only slightly from the untreated sample, with ±2%. A high whiteness index was obtained in the samples treated with only a binding agent; therefore, it can be concluded that the type of the resin used had no influence; the whiteness index loss of the cotton fabric was due to the MMT application.

To analyze the color obtained by the samples after each of the treatments, the chromatic coordinates *L, *a, and *b of the CIELAB color space are shown in Table 3.

The color difference calculated from the colorimetric parameters L, a, and b coincided with the results of the degree of whiteness, as the samples with the lowest degree of whiteness were the fabrics treated with MMT KSF, coinciding with the fabrics that showed the greatest color difference when compared with the results of the color measurement of the untreated sample.

Observing the value of the colorimetric parameters of the tissues treated with MMT KSF, it is the parameter *b that shows a significantly higher value than the results for the same parameter in the untreated sample. Increasing the value of *b meant that the sample acquired a yellowish color.

### 3.3. Ion Count

**Figure 4:**
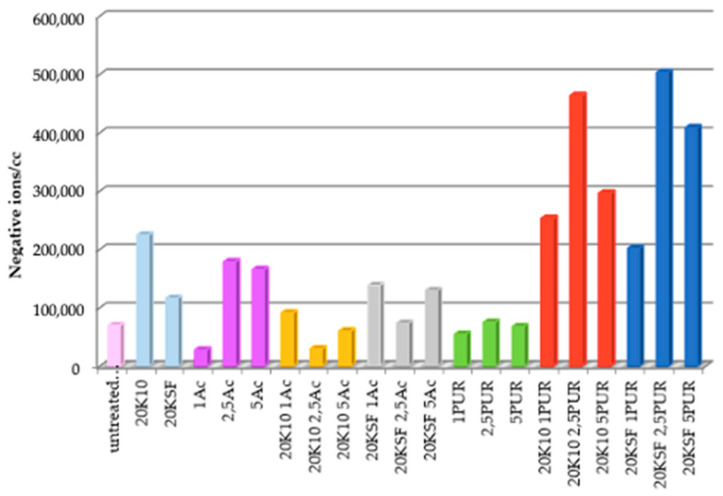
Average of total negative ions/cc in the air for 15 min.

Cotton fabrics treated with different formulations were tested in order to measure the amount of NAI released by the fabric. Figure 4 shows the average NAI realized from each sample during the tests for 15 min. Samples treated with only silicates show a significant difference between montmorillonite KSF and K10, with K10 reaching almost double in negative ion count compared to KSF. This could be due to the large surface area that MMT K10 possesses (220–270 m^2^/g).

While the samples treated with both silicates and polyurethane binder reached negative ion totals nearly five times the number generated by an untreated sample, fabrics treated with both silicates and acrylic binder did not surpass the negative ion count achieved by the silicates without binder.

**Figure 5:**
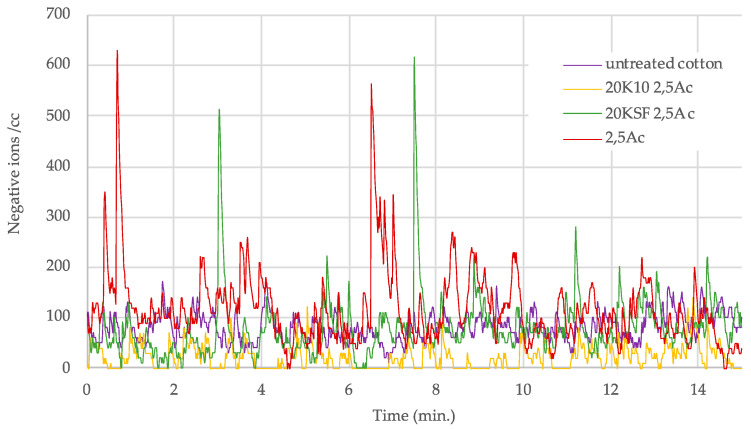
Negative ions/cc in 15 min released by treated samples with 2.5 g/L of acrylic binder.

**Figure 6:**
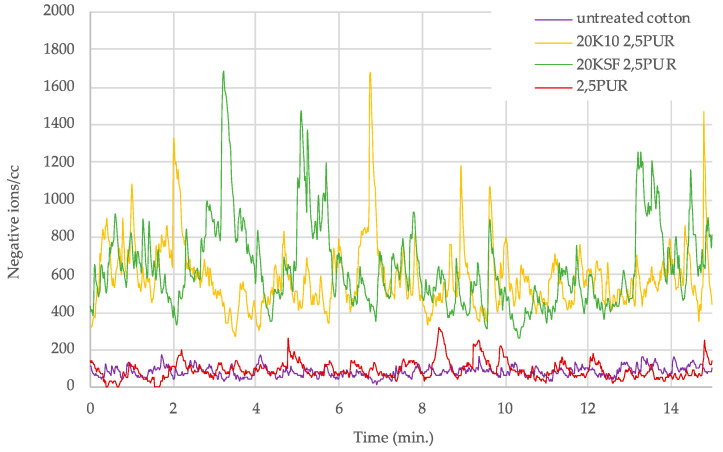
Negative ions/cc in 15 min released by treated samples with 2.5 g/L of polyurethane binder.

A clear trend could be seen in the samples treated with both MMT and the polyurethane (PU) binder, with optimal ion count at a binder concentration of 2.5 g/L. Figure 5 and Figure 6 show a graph with the number of negative ions measured for 15 min, comparing the behavior of untreated cotton and treated cotton using 20 g/L each of MMT, KSF, and K10, and with only 2.5 g/L of resin. In Figure 5 are presented the results of the acrylic binder, and Figure 6 shows the number of negative ions in the treated samples with polyurethane resin.

Figure 6 shows a clear separation between the ion count of the fabric treated with both silicate and PU resin in comparison to the untreated sample and the sample treated with only PU binder. The ion counts for the untreated samples and those treated PU binder only rarely increased above 200 ions, while the samples treated with both the silicate and PU binder remained mainly in the range between 400 and 1000 ions/cc, with a few peaks above 1000 ions/cc.

As the optimal ion count was achieved with a PU binder concentration of 2.5 g/L, an analysis of the surface areas was carried out using FESEM.

**Figure 7:**
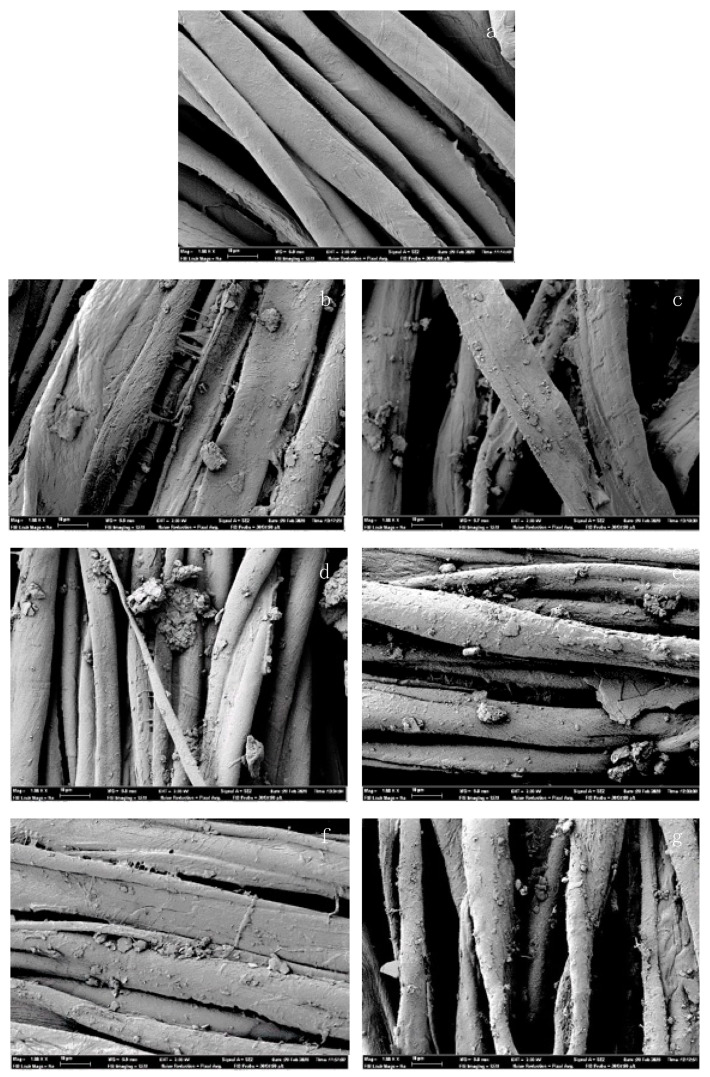
SEM images of samples 1000× (**a**) untreated cotton; (**b**) 20KSF; (**c**) 20K10; (**d**) 20KSF 2.5 Ac; (**e**) 20K10 2.5 Ac; (**f**) 20KSF 2.5 PUR; (**g**) 20K10 2.5 PUR.

In order to compare the deposition of the particles on the textile, a characterization of the fabrics treated with both MMT particles and using 2.5 g/L of both resins was carried out, and the images were compared with the microphotography of the untreated fabric. In Figure 7, the FESEM microphotographs of each analyzed sample are shown.

All SEM images of the treated fabrics verified the presence of micro-sized particles attached to the fiber surfaces of the fabrics treated with montmorillonite when compared to those of the untreated fabric (Figure 7a). The presence of resin in the fabrics treated with this auxiliary product, which helps to fix the particles to the surface of the fibers (Figure 7d–g), is evident.

The images in Figure 7b,c clearly show the difference between the two silicates in terms of particle size, while Figure 7b shows a sample treated only with KSF (20 g/L), where the particles were attached to the cotton fibers. The particles seen in (b) were significantly larger than those seen in (Figure 7c), a sample treated only with K10 (20 g/L); this was a logical consequence of their particle size ranges, which were 1–90 µm and 1–40 µm, respectively.

The images in Figure 7d,f show that more montmorillonite particles were present on the fiber surfaces of the binder-treated fabrics. However, on the surfaces of the fibers of the fabrics treated without binder, as shown in Figure 7b,c, there was only a small amount of widely dispersed montmorillonite. Therefore, the binder can be considered as a medium that enhances the attachment of the montmorillonite particles to the cotton fiber surface.

The images in Figure 7b,e do not show the same homogeneous dispersion as Figure 7f,g. Figure 7d shows an accumulation of particles, suggesting that the KSF was not compatible with the acrylic coating agent, as it did not disperse the silicate particles homogeneously throughout the cotton fiber, as observed in Figure 7f,g. Although the samples treated with KSF and acrylic binder showed a weakened ion count related to the increased binder concentration, these results did not differ from the ion count achieved by the sample treated with K10 and acrylic binder significantly enough to warrant further analysis.

Some literary sources suggest an explanation for the homogeneous dispersion of MMT particles and the strong binding properties exhibited by MMT when combined with the PUR binder. According to the work reported by authors Thomas et al. [19], the MMT-PUR matrix depends mainly on the interfacial interaction between the polar groups present on the silicate surface and the amino and carbonyl groups of the polyurethane bonds (-OOC-NH-) in the PUR matrix.

## 4. Discussion

The results showed a clear difference between the nature of the resin agent used and the ions released by the treated fabric. In the SEM images showed in Figure 7, the fabrics treated with acrylic resin showed a greater agglomeration of the acrylic particles, while the distribution of particles in the textile was less uniform. Moreover, the alkylammonium from the PUR exchange reaction with montmorillonite is something to take into consideration when trying to explain the significant results achieved by the samples treated with both MMT and PUR binder. This phenomenon concerns the cation exchange between the alkylammonium and the cations present on the surface and in between the layers of phyllosilicates.

The alkylammonium cations bind to the charged silicate surface by electrostatic bonding, although additional physical, non-Coulombic forces can also cause the adsorption of alkylamine. Van der Waals forces between the aliphatic residues and the surface, as well as between adjacent molecules or ions, contribute to the adsorption forces and become progressively significant when the molecular weight increases [20]. Therefore, the adsorption of alkylammonium originating from the linear polymeric polyurethane chains is irrevocably high and exceeds the CEC of the MMT.

It has also been concluded that a positive correlation exists between CEC and surface potential [21], so the increase in CEC and subsequent rise in surface potential could be why samples treated with both silicate and PUR binder achieve such remarkable results.

## 5. Conclusions

The results showed a significant difference in the number of negative ions released from the textile, depending on the type of resin used in the treatment with two types of MMT particles. The experimental results, taken by ION COUNTER COM-3200cd and ZEISS AURIGA Compact model FESEM, show that the best results were accomplished with 20 g/L MMT KSF and 2.5 g/L PUR binder concentration. As for the change in appearance produced by the color difference, it is clear that if MMT particles are used to functionalize the textile, the white fabric tends to fade, thus losing whiteness.

## Figures and Tables

**Table 1:** Characteristics of silicate particles used.

Silicate Particle	Size Range (µm)	Surface Area (m^2^/g)
MMT K10	1–40	220–270
MMT KSF	1–90	20–40

**Table 2:** Formulation used in each fabric treatment.

Sample	MMT	MMT Concentration (g/L)	Binder	Binder Concentration (g/L)
1 PUR	-	-	Polyurethane	1
2.5 PUR	-	-	Polyurethane	2.5
5 PUR	-	-	Polyurethane	5
20K10 1 PUR	K10	20	Polyurethane	1
20K10 2.5 PUR	K10	20	Polyurethane	2.5
20K10 5 PUR	K10	20	Polyurethane	5
20KSF 1 PUR	KSF	20	Polyurethane	1
20KSF 2.5 PUR	KSF	20	Polyurethane	2.5
20KSF 5 PUR	KSF	20	Polyurethane	5
1 AC	-	-	Acrylic	1
2.5 AC	-	-	Acrylic	2.5
5 AC	-	-	Acrylic	5
20K10 1 AC	K10	20	Acrylic	1
20K10 2.5 AC	K10	20	Acrylic	2.5
20K10 5 AC	K10	20	Acrylic	5
20KSF 1 AC	KSF	20	Acrylic	1
20KSF 2.5 AC	KSF	20	Acrylic	2.5
20KSF 5 AC	KSF	20	Acrylic	5

**Table 3:** CIELAB color space colorimetric parameters of the treated samples.

	*L	*a	*b	ΔEab
Untreated cotton	92.6151	−0.3076	3.6025	
1 PUR	92.8554	−0.3019	3.2629	0.4161
2.5 PUR	92.8842	−0.2419	3.3100	0.4028
5 PUR	92.9384	−0.3024	2.9786	0.7027
20K10 1 PUR	92.7023	−0.2957	4.6318	1.0331
20K10 2.5 PUR	92.4468	−0.2773	3.9199	0.3598
20K10 5 PUR	92.4668	−0.2209	4.0816	0.5090
20KSF 1 PUR	89.6774	0.1940	8.0724	5.3723
20KSF 2.5 PUR	90.2379	0.0954	7.1682	4.3044
20KSF 5 PUR	90.6665	0.1442	7.8069	4.6560
1 Ac	92.7645	−0.2082	3.3649	0.2977
2.5 Ac	92.9828	−0.3589	3.1566	0.5802
5 Ac	92.6826	−0.3038	3.1462	0.4613
20K10 1 Ac	92.3274	−0.1925	4.1027	0.5884
20K10 2.5 Ac	92.4160	−0.2220	4.2551	0.6876
20K10 5 Ac	92.9831	−0.2681	4.0742	0.5996
20KSF 1 Ac	90.6885	0.1031	7.7154	4.5603
20KSF 2.5 Ac	90.1750	0.0971	7.7494	4.8285
20KSF 5 Ac	90.6885	0.1003	7.6528	4.5037
20K10	92.4903	−0.2288	4.3796	0.7910
20KSF	90.8828	0.1081	7.5836	4.3615
